# Peroral choledochoscope-assisted removal of residual guidewire embedded in the mucous membrane of the pancreatic duct

**DOI:** 10.1055/a-2271-3944

**Published:** 2024-03-08

**Authors:** Yaoting Li, Lichao Zhang, Baoru Zhang, Tingting Yu, Yankun Hou, Jiao Tian, Senlin Hou

**Affiliations:** 1 Department of Biliopancreatic Endoscopic Surgery, The Second Hospital of Hebei Medical University, Shijiazhuang, China; 2 Gastroenterology Department, The 981st Hospital of PLA, Chengde, China

A 36-year-old man underwent endoscopic retrograde cholangiopancreatography (ERCP) at a local hospital for chronic pancreatitis, after which the broken end of a guidewire remained in the pancreatic duct. The patient had intermittent postoperative abdominal discomfort and was referred to our hospital.

The patient underwent ERCP, and fluoroscopy showed the residual guidewire in the pancreatic duct ( Fig. 1 **a** ). Neither a basket nor balloon could successfully remove the residual guidewire from the pancreatic duct. Subsequently, a peroral choledochoscope was used to explore the pancreatic duct, and revealed that the guidewire was embedded in the mucosa of the pancreatic duct and could not be removed ( Fig. 1 **b** ). We inserted a guidewire into the gap between the residual guidewire and the pancreatic duct mucosa with assistance from the choledochoscope ( Fig. 2 , Video 1 ). Then, we inserted a balloon along the guidewire and inflated the balloon when it entered the gap ( Fig. 3 ). On reinsertion of the choledochoscope, both ends of the guidewire could be seen ( Fig. 4 **a** ). The guidewire was removed with a net basket under direct view through the choledochoscope ( Fig. 4 **b** , Fig. 5 ).

**Fig. 1:**
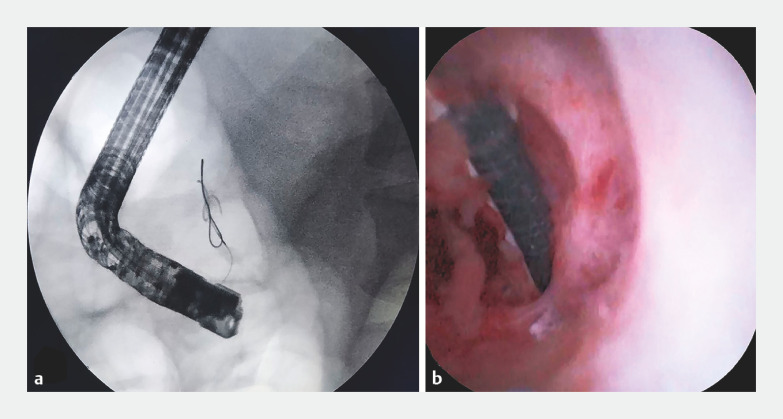
Visualization of the guidewire. **a** The residual guidewire was observed in the pancreatic duct under X-ray. **b** Peroral choledochoscope exploration of the pancreatic duct showed the residual guidewire embedded in the mucosa of the pancreatic duct.

**Fig. 2:**
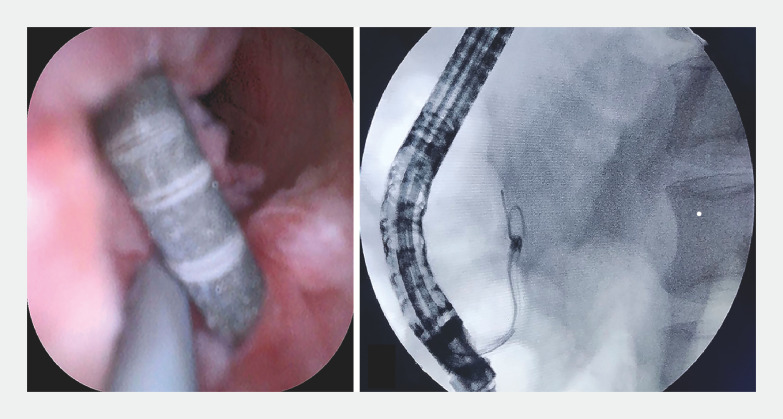
Preparing to retrieve the residual guidewire. **a,b** The guidewire was inserted into the gap between the residual guidewire and the pancreatic duct mucosa with the assistance of a transoral choledochoscope.

**Fig. 3:**
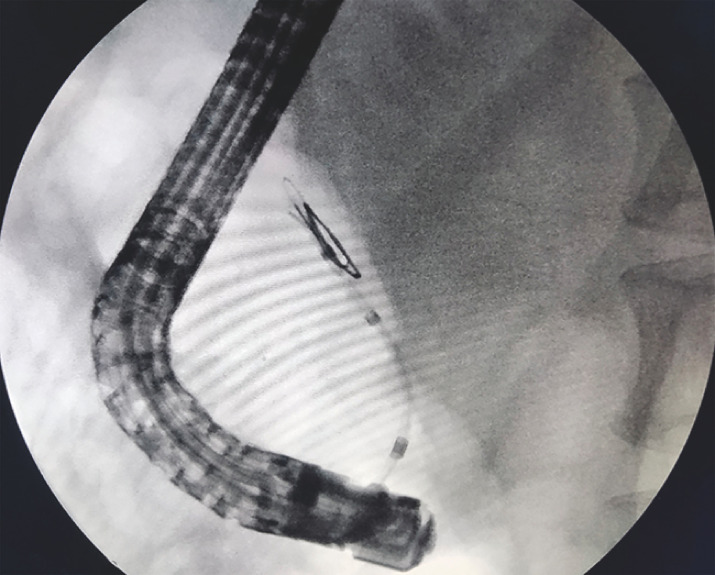
A balloon was inserted along the guidewire into the gap formed by the residual guidewire and the mucous membrane of the pancreatic duct.

**Fig. 4:**
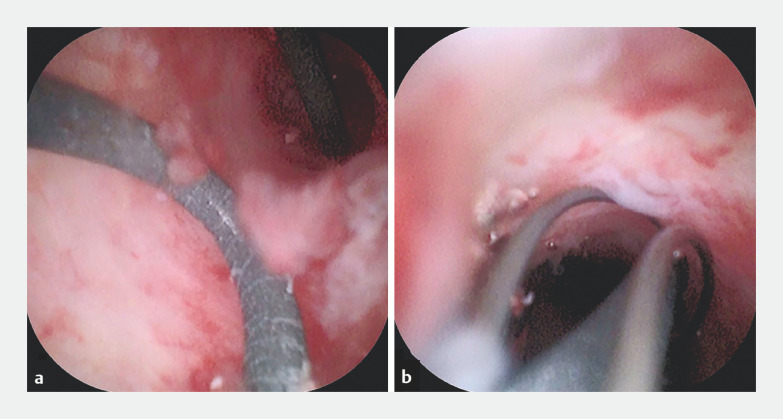
Retrieval of the guidewire. **a** Both ends of the guidewire protruded from the mucous membrane of the pancreatic duct. **b** A net basket was used to retrieve the residual guidewire.

**Fig. 5:**
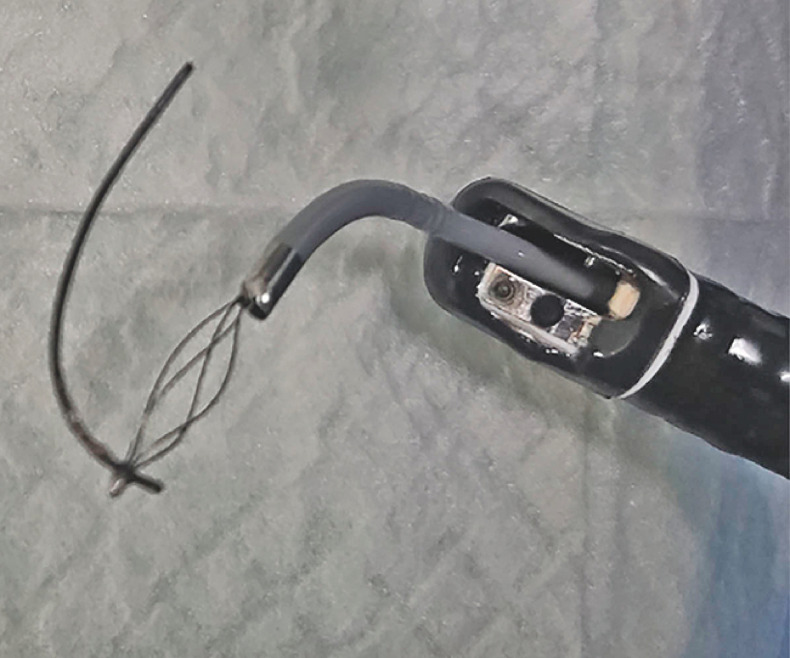
The guidewire was removed from the body.

The residual guidewire embedded in the mucous membrane of the pancreatic duct was removed with the assistance of a peroral choledochoscope.Video 1

Guidewire fragments left in the pancreatic duct are rare and usually occur in the treatment of complex diseases such as chronic pancreatitis 1 . This increases the risk of pancreatitis flare-ups and perforations. Surgical treatment is required when the fragment cannot be removed 2 . Endoscopic removal of residual guidewires is very challenging 3 4 . In our case, the residual guidewire was embedded in the mucous membrane of the pancreatic duct and could not be removed with conventional tools. The guidewire was removed from the mucosa by inserting a balloon into the gap and inflating it with the assistance of a transoral choledochoscope. This procedure offers a new option for endoscopic retrieval of foreign bodies in the pancreatic duct.

Endoscopy_UCTN_Code_TTT_1AR_2AK

## Notes

Endoscopy E-Videos https://eref.thieme.de/e-videos
*E-Videos* is an open access online section of the journal *Endoscopy* , reporting on interesting cases and new techniques in gastroenterological endoscopy. All papers include a high-quality video and are published with a Creative Commons CC-BY license. Endoscopy E-Videos qualify for HINARI discounts and waivers and eligibility is automatically checked during the submission process. We grant 100% waivers to articles whose corresponding authors are based in Group A countries and 50% waivers to those who are based in Group B countries as classified by Research4Life (see: https://www.research4life.org/access/eligibility/ ). This section has its own submission website at https://mc.manuscriptcentral.com/e-videos .
